# A Study of IFN-α-Induced Chemokines CCL2, CXCL10 and CCL19 in Patients with Systemic Lupus Erythematosu

**DOI:** 10.3390/life12020251

**Published:** 2022-02-08

**Authors:** Mariela Gencheva Geneva-Popova, Stanislava Dimitrova Popova-Belova, Petya Nikolova Gardzheva, Krasimir Iliev Kraev

**Affiliations:** 1 Department of Propedeutics of Internal Disease, Faculty of Medicine, Medical University-Plovdiv, “Vasil Apriliv” blvd 15-A, Rheumatology, UMHAT “St. Georgi”, 4002 Plovdiv, Bulgaria; Stanislva.Popova@mu-plovdiv.bg; 2 Department of Microbiology and Immunology, Faculty of Pharmaceutical Medicine, Medical University-Plovdiv, “Vasil Apriliv” blvd 15-A, Microbiology, UMHAT “St. Georgi”, 4002 Plovdiv, Bulgaria; petya.gardzheva@mu-plovdiv.bg; 3 Department of Propedeutics of Internal Disease, Faculty of Medicine, Medical University-Plovdiv, “Vasil Apriliv” blvd 15-A, Rheumatology, UMHAT “Kaspela”, 4002 Plovdiv, Bulgaria; Krasimir.Kraev@mu-plovdiv.bg

**Keywords:** IFN-α-induced chemokines, CCL2, CXCL10 and CCL19, SLE

## Abstract

The role of IFN-α-induced chemokines CCL2, CXCL10 and CCL19 in different forms of SLE has not been studied in Bulgaria, with worldwide sources attributing varying degrees of importance. The aim of this study was to investigate the correlation between IFN-induced chemokines CCL2, CXCL10 and CCL19 and disease activity in patients with SLE over 24 months. Materials and methods: This study used data from 70 patients with SLE (age range 24–62 years) and a control group of 30 healthy volunteers matched for age and gender. Levels of chemokines CCL2, CXCL10 and CCL19 in lupus patients’ serum were measured by ELISA. The study examined clinical and clinical laboratory indicators, as well as measures of disease activity developed for lupus patients (SLEDAI and SLICC). Statistical program SPSS, Version 26 were used for statistical data processing with *p* < 0.05. At 24 months of follow-up, 12 patients were with deterioration, and they had an IFN-a of 363.76 ± 9.23 versus 116.1 ± 22.1 pg/mL of those who did not worsen, CCL2 278.3 ± 5.12 versus 89.4 ± 12.8, CXCL10 234.2 ± 6.13 versus 115.23 ± 5.9 p CCL19 776.25 ± 5.1 vs. 651.34 ± 9.0 during the first visit. Results: The mean values of CCL2, CXCL10 and CCL19 were higher in patients with SLE compared to healthy controls (*p* = 0.01). A strong significant association (*p* = 0.01) was found between the concentration of CCL2, CXCL10 and CCL19 and with patients’ age, disease duration, SLEDAI and SLICC. Conclusion: CCL2, CXCL10 and CCL19 serum levels were found to correlate with patients’ age and disease duration. The level of IFN-induced chemokines CCL2, CXCL10 and CCL19 has a prognostic value in terms of SLE disease activity and degree of organ damage.

## 1. Introduction

Systemic lupus erythematosus (SLE) is a chronic, multisystem autoimmune disease characterized by exacerbations and remissions. The clinical manifestations of SLE vary in severity and include life-threatening renal, cardiac and hematological pathology, as well as secondary complications [1,2,3]. A hallmark and most characteristic feature of SLE is abnormally stimulated B-cell function, accompanied by hypergammaglobulinemia and the production of autoantibodies which bind to circulating nuclear antigens [4]. There is a strong association between the presence of antibodies to double-stranded DNA (anti-ds-DNA) and anti-ribonucleoprotein antibodies (anti-RNP) on one hand, and interferon (IFN)-α type I signalling pathway activity in patients with SLE on the other [5,6]. There is a specific IFN “gene signature” which increases the transcriptional activity of IFN-inducible genes in a large number of patients with SLE [7].

The main source of type I interferons is plasmacytoid dendritic cells (pDCs), which captures immune complexes containing DNA/RNA via the Fc gamma receptor IIA (CD32A). DNA/RNA capture activates intracellular Toll-like receptors (TLRs), particularly TLR7 and TLR9, which have an affinity for nucleic acids. Activation of TLRs triggers the production of type I interferons [5].

The type I interferon family includes 13 IFN-α subtypes, IFN-β, IFN-delta-δ, IFN-ε, IFN-κ and IFN-ο. IFN type I and both IFN-α receptors (IFNAR1 and IFNAR2) form a functional IFNAR complex, leading to phosphorylation of STAT1 and STAT2 [8,9].

The activation of the IFNAR complex mediates the induction of ISRE to stimulate the production of larger amounts of IFN type I, and this leads to amplification of the interferon response [9].

In autoimmune diseases, immune cells tend to produce large amounts of IFN at inflammation sites in a short period of time, which leads to tissue damage and deterioration of the patient’s condition [2,3]. Type I IFN’s functions listed here and the impaired clearance of apoptotic particles (residues) in patients with SLE promote the formation of immune complexes that are potent inducers of IFN type I. Thus, inadequate IFN production and/or inability to suppress the effects of signalling pathway activation can initiate a positive feedback loop, leading to the persistence of the autoimmune response [10,11].

The results of studies conducted with patients [8,9] are in support of the detrimental effects of IFN-I, especially when its concentrations are high over a long period of time. The increased activity of an unestablished and unidentified substance in the serum of a lupus patient which leads to increased stimulatory function of T cells in an allogeneic reaction system with a mixed composition of lymphocytes is inhibited by anti-IFN-α antibodies. Therefore, it can be assumed with a certain degree of likelihood that IFN-alpha promotes the activation of autoreactive T cells [8]. IFN-α induces the production of the B lymphocyte stimulator (BLyS), thus facilitating B-cell differentiation as well as stimulating the switching of immunoglobulin classes, with the end result being the generation of potentially pathogenic autoantibodies [4].

There is ample evidence from previous studies supporting the pathogenic role of IFN-α in target organs, which is associated with SLE patients’ morbidity and mortality [12]. pDCs predominate in kidney biopsy material obtained from patients with membrane-proliferative glomerulonephritis, with IFN-alpha transcripts also present in the biopsy material. IFN-α also damages podocytes and induces the production of chemokines, which are responsible for the recruitment of inflammatory cells (most notably neutrophils) in the kidneys and other affected tissues [12]. In this regard, high levels of expression of IFN-induced chemokines could make them a future biomarker predicting impending lupus exacerbation [12].

The stimulated production of chemokines is an important link between the activation of the IFN type I signalling pathway and systemic lupus [13]. Chemokines regulate leukocyte migration throughout the body thus directing the immune response. IFN-type-dependent stimulated chemokine production may lead to inadequate recruitment of autoreactive lymphocytes at inflammation sites [14]. Systemic elevations in chemokine levels may desensitize chemokine receptors on the surface of activated lymphocytes, leading to loss of normal defence mechanisms and the subsequent development of a large-scale inflammatory response.

Although the exact mechanism is unknown, IFN-regulated chemokines may be markers of underlying pathophysiology in SLE [15,16,17].

Two of these chemokines show potential biomarkers in SLE. According to Dominguez–Gutierrez et al. publication, these chemokines are CCL2 and CXCL10 [18].

CCL2, formerly known as monocyte chemotaxis protein-1 (MCP-1), is a potent recruiter of monocytes, T cells, basophils and dendritic cells at the site of infection or tissue damage, but it has no effect on neutrophils and eosinophils except unless the N-terminus of CCL2 is cut [19]. Some cell types, such as monocytes, macrophages and dendritic cells, may secrete CCL2 which exerts its effects through the membrane receptors CCR2 and CCR4 and whose production is stimulated by IFN-α and IFN-b [11,20,21]. CCL2 has a significant role in cleansing tissues from pathogens and is associated with some pathological processes. In a study examining the serological proteome in systemic lupus using antibodies, CCL2 was identified as one of the 12 proteins with increased concentration [22,23]. CCL2 has been shown to be one of three chemokines which elevated levels precede a period of lupus exacerbation, indicating that they would be good predictors of increased lupus activity [6].

CXCL10, also known as IFN-gamma-inducible protein 10 (IP-10), is a chemokine from the C-X-C-chemokine family. It is secreted by IFN-gamma-stimulated endothelial cells, fibroblasts and monocytes [24,25].

Similar to CCL2, CXCL10 is a potent attractant for monocytes, macrophages, T cells, NK cells, and dendritic cells at sites of tissue damage and infection. CXCL10 is a cytokine of which production is stimulated by interferon [24]. CXCL10 binds to its CXCR3 receptor and exerts its effects by activating the Jak/STAT signalling pathway. CXCL10 is highly expressed in many human diseases. It has been shown to be involved in the pathogenesis of three major groups of human infectious diseases, and inflammatory and autoimmune diseases [26]. In patients with SLE, serum CXCL10 levels are strongly elevated and correlate with disease activity levels [12]. According to Marie and Habib, the expression of messenger RNA for CXCR3 and CXCL10 in urine correlates with the presence of nephritis [12]. The expression is stimulated in patients with active lupus nephritis, while in healthy controls it is imperceptible [12]. The authors believe that the combined study of serum levels of CCL2 and CXCL10 may be useful as a predictive factor for future exacerbations of SLE [18].

CCL19 (Chemokine (C-C motif) ligand 19, MIP-3B) is also a potential biomarker indicating disease activity in SLE. According to Bauer et al. [27], serum CCL19 levels in patients with active SLE (defined as SLEDAI ≥ 6) were significantly higher than serum CCL19 levels in patients with inactive lupus (defined as SLEDAI ≤ 2). In the same publication, serum CXCL10 levels were reported to be significantly elevated in patients with active SLE [27].

Serum concentrations of IFN-α-induced chemokines are a replacement marker for the activation of the IFN type I system, which is strongly implicated in the pathogenesis of SLE [28]. IFN- α induced transcriptional genes studied in peripheral blood are currently the most commonly used parameter to analyse the involvement of the interferon system in patients with SLE. Nonetheless, the most comprehensive longitudinal studies to date do not show a strong correlation between IFN genes and disease activity [29].

A study by Connelly et al. [20] confirmed a longitudinal relationship between the serum concentration of IFN type I-induced chemokines and SLE disease activity as measured by SLEDAI. Chemokines induced by IFN type I were found in 100% of patients, unlike many other cytokines, which are found in only a fraction of patients [20]. Data from this study reveal the existence of subgroups of patients with a widely variable degree of concordance between IFN-CK score and SLEDAI.

Serum chemokine levels have not been studied in a large group of patients with systemic lupus erythematosus.

The aim of this study was to investigate the correlation between IFN-α-induced chemokines CCL2, CXCL10 and CCL19 and disease activity in patients with systemic lupus erythematosus.

## 2. Materials and Methods

Between 2019 and 2021, this study analysed data from 70 patients (12 men and 58 women) meeting diagnostic criteria for SLE (revised classification criteria for SLE (1982) [30], and adopted by the American College of Rheumatology (ACR) (1997)) [31], with a disease duration between 2 and 20 years, and mean age of 39.00 ± 13.18 (range 22–62 years), as well as 30 healthy individuals with a mean age of 40.00 ± 5.67 (range 31–57 years). Patients with SLE were followed up for at least 24 months and were reviewed and examined at least every 6 months. At Visit Zero (month zero) and Visit One (6 months) 70 patients were examined, at Visit Three (12 months)—64 patients, at Visit Four (18 months) 61 patients and at Visit Five (24 months)—60 patients.

This study was performed in accordance with the principles of the Declaration of Helsinki. Study participants were provided written informed consent, detailed clinical questionnaire information. Researchers collected demographic information, blood samples and medical records.

A standard SLEDAI activity scale was used to assess disease activity [32,33]. In addition, we used the Injury Index in patients with systemic lupus (SLICC/ACR), which was developed to assess irreversible damage in patients with SLE, regardless of the cause [34,35].

The SLICC damage index (SDI) contains items which represent permanent, irreversible damage in a lupus patient. Items should be present for at least 6 months with the exception that manifestations such as myocardial infarction and stroke are recorded once they occur. Damage is defined for 12 organ systems: ocular (range 0–2), neuropsychiatric (0–6), renal (0–3), pulmonary (0–5), cardiovascular (0–6), peripheral vascular (0–5), gastrointestinal (0–6), musculoskeletal (0–7), skin (0–3), endocrine (diabetes) (0–1), gonadal (0–1), and malignancies (0–2). Damage over time can only be stable or increased, theoretically to a maximum of 47 points [34,35].

The study was approved by the ethics committee at the Medical University of Plovdiv. All participants gave informed consent before study enrolment. The theme of the project corresponds to the strategic directions of the scientific plan of MU-Plovdiv—Development of pharmacogenomics and personalized medicine.

## 3. Laboratory Analysis

The following parameters were determined: for inflammatory activity—C-reactive protein (CRP), erythrocyte sedimentation rate (ESR), fibrinogen, for autoimmune disorders–ANA, anti-ds-DNA, anti-Sm-Ab. Laboratory tests such as erythrocyte count, hemoglobin (Hb), leucocytes (leuc), trombocites (Trom) were performed using standard laboratory methods.

ELISA kits selected on the basis of their wide range of detection of tested antigens were included in the present study. These included the Human IFN-α (Interferon Alpha) ELISA Kit, catalogue № E-EL-H2532, detection range 15.63~1000 pg/mL sensitivity 9.38 pg/mL, Elabscience Biotechnology Inc., Houston, TX, USA, the Human CCL2 ELISA Kit, catalogue № E-EL-H0496 detection range 31.25~2000 pg/mL sensitivity 14.28 pg/mL, Elabscience Biotechnology Inc., USA, the Human CXCL10 ELISA Kit, catalogue №, E-EL-H0804 detection range 7.81~500 ng/mL pg/mL sensitivity 21.56 pg/mL, Elabscience Biotechnology Inc., USA and the Human CCL19 ELISA Kit, Catalog №, E-EL-H124-9 detection range 27.87~1000 pg/mL sensitivity 19.38 pg/mL, Elabscience Biotechnology Inc., USA. In the study, the patient’s serum was analyzed, and the dilution was 1:2—one part serum and two parts diluent whale. The kit only caught IFN I. The kit manufacturer did not report cross-reactivity.

Two primary antigen-specific antibodies were used for the assay. The first specific antibody was loaded by the manufacturer onto the microplate which was assembled from 12 separate strips of eight wells. After determining the number of standard and control samples required, the necessary number of strips was removed from the aluminum packaging while the rest were frozen at −20 C. In line with the manufacturer’s instructions, the lyophilized standard was diluted, and the necessary standard and negative control samples were prepared. The second specific antibody (Biotin-conjugated) and the peroxidase conjugated secondary antibody were diluted 1:100 with their respective diluents. After reading the optical density, the duplicate values of the standard and tested samples were averaged. Curve Expert 4 software was used to prepare the standard curve. After preparing the standard curve, the concentration of each sample was calculated, and the dilution factor of the sample was applied. All samples were prepared twice, according to the manufacturer’s requirements.

Anti-dsDNA is reported in IU/mL with a manufacturer-specified positive cut-off of 10.0 IU/mL, and other specificities as an Antibody Index (AI) value (range 0–8) based on the fluorescence intensity of each of the other autoantibody specificities, with a manufacturer-recommended positive cut-off of AI = 1.0.

Inclusion Criteria:

1. Patients with proven systemic lupus erythematosus (covering ACR criteria)
2. Patients with systemic lupus erythematosus (covering ACR criteria) on treatment with stable doses of corticosteroids, belimumab, DMARDs (hydroxychloroquine, methotrexate, leflunomide);
3. Patients who have capacity and no mental health comorbidities;
4. Patients who consented to participate by signing an informed consent form.

Exclusion Criteria:

1. Patients who refused to give informed consent;
2. Patients diagnosed with a rheumatic disease other than SLE;
3. Patients with decompensated cardiovascular, pulmonary or renal failure;
4. Pregnant or lactating women.

## 4. Statistical Analysis

Statistical analysis was performed using SPSS version 26.0 (SPSS Inc., Chicago, IL, USA). The concentrations of the parameters of iron homeostasis, inflammation and autoimmune disorders were tested for normality with the Kolmogorov–Smirnov test. Data were given as mean ± standard deviation (SD). The *t*-test was used to compare two groups with normal distribution and the Mann–Whitney U test was used to compare groups with non-normal distribution. Correlations between data were evaluated by calculating the Pearson’s correlation coefficient depending on the distribution of the continuous variables. *p* < 0.05 was considered as statistically significant.

Results

The study involved 70 patients with systemic lupus erythematosus. There was no difference in age between the SLE patient group (*t* = 5.06, *p* < 0.001) and the control group of healthy volunteers. The SLE group and the control group had a similar gender distribution (ϰ^2^ = 0.211; *p* = 0.406), with the number of women being 82.2% and 80%, respectively.

All patients, suffering from SLE received therapy as follows: DMARDs (*n* = 23), DMARDs and corticosteroids (*n* = 14), biological agents (Belimumab), DMARDs and corticosteroids (*n* = 31), corticosteroids (*n* = 2).

Table 1 shows the comparison between chemokines and immunological parameters in patients with SLE and healthy controls. The INF-a, CCL2, CXCL10, CCl19 levels were significantly higher in the SLE group than in the control group.

Patients with SLE were divided into two groups—group A—SLEDAI score below 8 (low disease activity) and—group B—SLEDAI score over 8 (high disease activity).

Table 2 presents a comparison between the tested chemokines levels and some indicators of inflammation according to disease activity as assessed by the SLEDAI disease activity index. The levels of the studied indicators IFN-α pg/mL, CCL2 pg/mL, CXCL10 pg/mL, CCL19 pg/mL are significantly higher in the group with activity compared to that without activity.

The effects of organ damage are reflected in the SLICC index and it gradually increases during the 24-month follow-up period (Table 3).

There is a significant correlation between the level of IFN-α pg/mL and the levels of the chemokines CCL2 pg/mL, CXCL10 pg/mL, CXCL19 pg/mL (*p* = 0.001).

Out of the 70 SLE patients in this study, 12 (17.14%) deteriorated during the 24-month follow-up period. Out of these 12 patients, three (25%) developed proteinuria over 3.5 g in 24 h and showed elevated levels of urea, creatinine, uric acid without the need for hemodialysis; another three (25%) developed pain, fatigue and joint swelling accompanied by increased butterfly face rash, and the remaining six (50%) demonstrated deterioration of hematological parameters. In these patients, the serum values of the tested chemokines at Visit Zero were significantly higher compared to all other patients with SLE (Table 5).

## 5. Discussion

The results from a study by Bauer et al. show that asymptomatic patients with systemic lupus (low-activity patients) with high serum chemokine levels are more vulnerable to exacerbation in the next year than patients with low serum chemokine levels [27]. The frequency of exacerbations for the group of patients with intermediate levels of chemokines is identical to that for the group of patients with low levels of chemokines. According to the authors, the increased risk of exacerbation is limited to the group of patients with significantly higher serum chemokine levels compared to controls [27]. Some of the patients with low chemokine levels at baseline worsened the following year, but most of these patients had progressively increasing chemokine concentrations the closer they became to exacerbations.

The results of Bauer et al. correspond to our findings, according to which the levels of the tested CCL2 pg/mL, CXCL10 pg/mL, CXCL19 pg/mL are significant compared to the levels of the control subjects.

Connelly et al. found that an increase in the activity of the IFN type I system was associated with an increase in SLEDAI, and this interdependence remained significant in a multivariate analysis taking into account other variables related to disease activity [20]. This is in support of previous studies by Bauer et al., which found that patients with high concentrations of IFN-induced chemokines at baseline were more vulnerable to exacerbations within the following year, and that IFN-CK scores increased in moments of exacerbation [27].

In studies conducted by Petri et al. investigating IFN-induced gene transcripts, there was an inversely proportional relationship between serological markers of disease activity, such as complement fragments (C3 and C4) and the degree of IFN type I activity in SLE [36,37,38]. The biological rationale for this relationship is related to the activation of the IFN type I system by immune complexes, neutrophilic traps or other endogenous stimuli in SLE. According to a study by Bennett et al. [39], Interferon type I transcriptional signature is also strongly associated with renal and, to a lesser extent, CNS and haematological manifestations of SLE.

We divided our participants into two groups according to their SLEDAI disease activity index and found that the levels of CCL2 pg/mL, CXCL10 pg/mL, CXCL19 pg/mL were significantly higher in the group with higher disease activity (SLEDAI score above 8) compared participants with SLEDAI score below 8, which is in keeping with other authors’ findings [20].

We checked patients’ levels of CCL2 pg/mL, CXCL10 pg/mL, CXCL19 pg/mL every 6 months and found that the level of IFN-a significantly decreased compared to baseline, while the levels of chemoks were significantly higher. Higher levels of CCL2 pg/mL, CXCL10 pg/mL, CXCL19 pg/mL throughout the follow-up period correlated with the SLICC index value. This suggests that frequent monitoring of chemokine levels would be helpful in identifying patients who are progressing towards disease exacerbation. Considering that the difference in frequency of exacerbations in patients with high chemokine levels compared to those with low levels occurs significantly as soon as the 100th day from baseline, it could be beneficial to measure chemokine levels even at narrower intervals [20]. Data from the same study indicate that routine laboratory markers are not good predictors of exacerbation of SLE and that they do not contribute to increasing the predictive value of chemokines in multivariate assays [20].

Monitoring serum chemokine levels in patients with SLE has emerged as an important tool in the hands of clinicians to assess the likelihood of future exacerbations of clinically inactive patients with SLE [27].

Markers linking interferon system activity to disease activity may be useful in identifying patients suitable for treatment with newly developed drugs that suppress the IFN type I signalling pathway.

## 6. Conclusions

1. CCL2, CXCL10 and CCL19 serum levels correlate with patients’ age and disease duration. The mean values of CCL2, CXCL10 and CCL19 were higher in patients with SLE compared to healthy controls (*p* < 0.01). A strong significant association (*p* = 0.001) was found between the concentration of CCL2, CXCL10 and CCL19 and disease activity measures SLEDAI and SLICC.
2. The level of IFN-induced chemokines (CCL2, CXCL10 and CCL19) has a prognostic value in terms of SLE disease activity and degree of organ damage.
3. CCL2, CXCL10 and CCL19 chemokines can be used as biomarkers for systemic lupus activity.

## Figures and Tables

**Table 1:** Comparison between chemokines and immunological parameters in patients with SLE and healthy controls (x ± SD).

Parametres	SLE *n* = 70	Controls *n* = 30	P1
IFN-α pg/mL	163.91 ± 7.16	42.1 ± 5.23	0.01
CCL2 pg/mL	179.22 ± 41.81	11.67 ± 4.18	0.01
CXCL10 pg/mL	45.24 ± 1.04	15.95 ± 1.96	0.01
CCL19 pg/mL	531.25 ± 109	22.34 ± 4.98	0.01
Hg	113.81 ± 5.87	145.9 ± 9.23	0.01
Leuc	5.87 ± 2.67	9.34 ± 4.98	NS
Trom	255.9 ± 33.23	267.9 ± 10.9	NS
CRP	52.34 ± 4.98	6.89 ± 4.12	0.01
ESR	40.87 ± 8.67	10.33 ± 2.98	0.01
ANA pos	70 (100%)	1 (3.33%)	0.01
Anti-ds-DNA-pos	58 (82.85%)	0	0.01
Anti-Sm- pos	45 (64.28)	0	0.01

**Table 2:** Comparison between chemokines and immunological parameters in patients with SLE as measured by the SLEDAI disease activity index ((x ± SD).

Parametres	SLE, SLEDAI < 8, *n* = 29	SLE, SLEDAI ≥ 8, *n* = 41	P1
IFN-α pg/mL	63.76 ± 4.23	204.1 ± 18.1	0.01
CCL2 pg/mL	41.67 ± 4.18	131.45 ± 27.83	0.01
CXCL10 pg/mL	29.24 ± 4.12	115.23 ± 5.22	0.01
CCL19 pg/mL	231.25 ± 56.8	782.34 ± 11.93	0.01
Hg	121.51 ± 6.89	104.2 ± 4.11	0.01
Leuc	5.87 ± 1.67	9.34 ± 1.16	0.05
Trom	301.2 ± 9.44	139.9 ± 10.2	0.05
CRP	19.9 ± 4.31	56.44 ± 4.83	0.01
ESR	21.33 ± 6.24	40.77 ± 3.81	0.01
ANA pos	29 (100%)	41 (100%)	NS
Anti-ds-DNA -pos	22 (75.86%)	36 (87.80%)	0.05
Anti-Sm- pos	13 (44.82%)	32 (78.04%)	0.01

**Table 3:** Comparison between chemokines and immunological parameters in patients with SLE according to the change of the SLICC index.

Parametres	SLE, *n* = 70 Month 0	SLE, *n* = 70 Month 6	SLE, *n* = 64 Month 12	SLE, *n* = 61 Month 18	SLE, *n* = 60 Month 24
IFN-α pg/mL	163.91 ± 7.16	154.1 ± 8.12	194.2 ± 3.89	157.2 ± 3.33	130.9 ± 4.3 *
CCL2 pg/mL	179.2 ± 41.81	201.6 ± 30.1	210.8 ± 9.8	279.4 ± 21.9	192.2 ± 14.5 **
CXCL10 g/mL	45.24 ± 1.04	57.4 ± 2.08	65.3 ± 7.99	69.9 ± 2.67	71.78 ± 2.1 **
CCL19 pg/mL	531.25 ± 109	543.3 ± 89.3	682.5 ± 11.4	567.5 ± 71.2	604.2 ± 87.7 **
SLEDAI	11.67 ± 2.34	14.2 ± 1.34	16.76 ± 3,2	17.1 ± 3.81	14.1 ± 4.12 *
SLICC	13.78 ± 2.1	19.94 ± 1.1	22.4 ± 1.3	23.1 ± 2.7	25.2 ± 1.4 **

* A significant reduction in the value of the studied indicator compared to the first study. ** A significant increase in the value of the studied indicator compared to the first study. The analysis of the examined chemokines shows the presence of significant correlations between their level and both the SLEDAI disease activity index and the SLICC disease damage index (Table 4).

**Table 4:** Correlations between chemokine levels and SLEDAI and SLICC disease indices in patients with SLE.

Parameters	Patients with SLE, Month 0, Visit 1			Patients with SLE, Month 12, Visit 3			Patients with SLE, Month 24, Visit 5		
	*n*	*r*	*p*	*n*	*r*	*p*	*n*	*r*	*p*
IFN-α/SLEDAI CCL2/SLEDAI CXCL10/SLEDAI CCL19/SLEDAI CRP/SLEDAI ESR/SLEDAI	70	0.319 0.341 0.299 0.266 0.389 0.401	0.001 0.027 0.013 0.016 0.024 0.001	60	0.322 0.389 0.306 0.296 0.404 0.297	0.001 0.031 0.015 0.016 0.016 0.001	60	0.319 0.320 0.397 0.356 0.341 0.391	0.001 0.033 0.001 0.002 0.026 0.001
IFN-α/SLICC CCL2/SLICC CXCL10/SLICC CCL19/SLICC CRP/SLICC ESR/SLICC	70	0.420 0.345 −0.699 −0.615 −0.423 0.410	0.001 0.001 0.013 0.016 0.026 0.024	60	0.492 0.374 −0.642 −0.789 −0.423 0.309	0.001 0.001 0.013 0.015 0.001 0.021	60	0.429 0.320 −0.705 −0.789 −0.576 0.419	0.001 0.001 0.001 0.002 0.001 0.027
IFN-α/CCL2 IFN-α/CXCL10 IFN-α/CCL19 CCL2/CXCL10 CCL2/CXCL19 CXCL10/CXCL19	70	0.523 0.509 0.519 0.766 0.789 0.584	0.001 0.001 0.013 0.032 0.001 0.001	60	0.522 0.514 0.487 0.710 0.792 0.582	0.001 0.001 0.013 0.021 0.001 0.001	60	0.523 0.570 0.397 0.756 0.741 0.591	0.001 0.001 0.013 0.024 0.001 0.001

SLEDAI index correlated positively with IFN-α (*r* = 0.319, *p* = 0.001), CCL2 (*r* = 0.341, *p* = 0.027), CXCL10 (*r* = 0.299, *p* = 0.013), CCL19 (*r* = 0.266, *p* = 0.016). SLICC correlated negatively with CXCL10 (*r* = −0.699, *p* = 0.013), CCL19 (*r* = −315, *p* = 0.16) and SRS (*r* = −0.423, *p* = 0.026) on the first visit, and this trend continued throughout the follow-up period of 24 months CXCL10 (*r* = −0.705, *p* = 0.001), CCL19 (*r* = −615, *p* = 0.01) and SRS (*r* = −0.576, *p* = 0.01).

**Table 5:** Comparison between chemokines, haematological parameters and immunological parameters at the first visit in 70 patients with systemic lupus who deteriorated during 24 months of follow-up.

Parametres	SLE, Deterioration, *n* = 12	SLE, No Change, *n* = 58	P1
IFN-α pg/mL	363.76 ± 9.23	116.1 ± 22.1	0.001
CCL2 pg/mL	278.3 ± 5.12	89.4 ± 12.8	0.001
CXCL10 pg/mL	234.2 ± 6.13	115.23 ± 5.9	0.001
CCL19 pg/mL	776.25 ± 5.1	651.34 ± 9.0	0.001
Hg	110.51 ± 3.9	120.2 ± 9.1	0.001
Leuc	3.54 ± 1.27	9.99 ± 1.8	0.001
Trom	131.2 ± 9.78	186.9 ± 10.9	0.05
CRP	58.5 ± 4.31	32.6. ± 4.55	0.001
ESR	78.4 ± 6.99	40.21 ± 4.07	0.001
ANA pos	12 (100%)	58(100%)	NS
Anti-ds-DNA -pos	12 (100%)	46 (79.31%)	0.05
Anti-Sm- pos	12 (100%)	33 (56.89%)	0.001

## Data Availability

Data supporting the reported results can be found in the archives of St. George University Hospital, DCC St. George’s and the journals of the Immunology Laboratory, Department of Microbiology and Immunology, St. George University Hospital.

## Sample Availability

Samples of patients are available at the Department of Microbiology and Immunology, Medical University of Plovdiv, Bulgaria.

## Abbreviations

ACR	American College of Rheumatology
anti-RNP	anti-ribonucleoprotein antibodies
BLyS	B lymphocyte stimulator
CCL2	monocyte chemotaxis protein-1 (MCP-1)
CXCL10	IFN-gamma-inducible protein 10 (IP-10)
CCL19	Chemokine (C-C motif) ligand 19
ds	DNA double-stranded DNA ()
ELISA	enzyme-linked immunosorbent assay
IFN-α	Interferon α
IFNAR	IFN-α receptors
IRF	IFN-regulatory factor
ISRE	IFN-stimulated response element
pg/mL	Picograms per millilitre
pDCs	plasmacytoid dendritic cells
SLE	systemic lupus erythematosus
SLEDAI	Systemic Lupus Erythematosus Disease Activity Index
SLICC	Systemic Lupus International Collaborating Clinics
SPSS	Software Package Scientific Statistics
TLRs	Toll-like receptors
